# Use of digital patients in clinical simulation for nursing education: a scoping review protocol

**DOI:** 10.3389/fdgth.2026.1767182

**Published:** 2026-04-02

**Authors:** Bárbara Letícia de Queiroz Xavier, Joyanne de Souza Medeiros, Rayssa Horacio Lopes, Janmilli da Costa Dantas Santiago, Cristiane da Silva Ramos Marinho, Amanda Soares, Richardson Augusto Rosendo da Silva

**Affiliations:** 1 Collective Health Program in Collective Health, Federal University of Rio Grande do Norte, Natal, Brazil; 2 Library, Faculty of Health Sciences of Trairi, Federal University of Rio Grande do Norte, Santa Cruz, Brazil; 3 School of Health, Federal University of Rio Grande do Norte, Natal, Brazil; 4 Department of Nursing, Federal University of Rio Grande do Norte, Natal, Brazil; 5 Faculty of Health Sciences of Trairi, Federal University of Rio Grande do Norte, Santa Cruz, Brazil.; 6 Program in Collective Health, Department of Nursing, Federal University of Rio Grande do Norte, Natal, Brazil

**Keywords:** digital health, health, health informatics, nursing, simulation clinical

## Abstract

**Introduction:**

Clinical simulation represents an emerging educational technology delivered through software-based platforms, accessible via computers or head-mounted displays. It is characterized as a partially immersive, screen-mediated experience in which learners are placed in simulated roles that require the execution of psychomotor actions, clinical decision-making, and interpersonal communication skills.

**Methods and analysis:**

This scoping review protocol follows the methodological guidance of the Joanna Briggs Institute and adheres to the Preferred Reporting Items for Systematic Reviews and Meta-Analyses extension for scoping reviews (PRISMA-ScR). Comprehensive searches will be conducted across the following electronic databases: MEDLINE/PubMed, Embase, Web of Science, Education Resources Information Center (ERIC), and the Cumulative Index to Nursing and Allied Health Literature (CINAHL). In addition, gray literature sources will be explored through national and international repositories, including the Catalogue of Theses and Dissertations of the Coordination for the Improvement of Higher Education Personnel (CAPES), the Electronic Theses Online Service (EthOS), the Open Access Scientific Repository of Portugal (RCAAP), the National ETD Portal, Theses Canada, the Portal de Tesis Latinoamericanas, and WorldCat Dissertations and Theses. The review seeks to address the question: “What evidence exists regarding the use of clinical simulation with digital patients in the teaching and learning process of nursing students?” Eligible sources will include studies with full-text availability, encompassing peer-reviewed research articles, theses, dissertations, and other relevant documents, without restrictions related to geographic location, publication date, or language. Data will be charted using a customized extraction form based on Joanna Briggs Institute recommendations. Quantitative findings will be summarized using descriptive statistical methods, while qualitative evidence will be examined through thematic analysis. Ethical approval is not required.

**Ethics and disclosure:**

Given the methodological nature of this study, formal ethical approval is not required. The findings are intended for dissemination through publication in a peer-reviewed journal and presentation at scientific conferences. To promote transparency and reinforce the originality of the review, this protocol has been prospectively registered on the Open Science Framework (OSF).

**Clinical Trial Registration:**

Protocol registration in the open science framework (OSF): DOI: 10.17605/OSF.IO/GAXR6

## 1 Introduction

Virtual simulations (VS) are described as a promising type of educational technology that are run through software applications on computers or mounted displays. Within the context of nursing education, this technological approach enables students to rehearse clinical procedures in interactive digital environments that incorporate virtual spaces, instruments, and characters. These environments may include simulated patients and healthcare professionals supported by artificial intelligence, designed to replicate key aspects of real-world clinical practice (1).

VS are considered emerging technologies due to their functionality as part of the educational process. This makes it necessary for them to be implemented in the teaching curriculum, since they are still lacking (1) and evidence of their effectiveness is at an early stage (2). VS is characterized as a screen-mediated, partially immersive learning experience in which individuals are placed within simulated scenarios that require the execution of psychomotor tasks, clinical decision-making processes, and/or communication-related skills (3).

This simulation effectively provides education and assessment activities in various health disciplines to facilitate the development and refinement of clinical skills and work routines (4).

The provision of training within university settings has been constrained by limitations related to physical infrastructure and scheduling, as well as shortages of qualified personnel and contextual factors that hinder the delivery of authentic, practice-oriented learning experiences. Given this, VS can be used as one of the strategies for clinical practice in situations where students' access to the real field is impossible, as in the context of the COVID-19 pandemic, where it was necessary to develop effective methods, since it has been considered a successful strategy for promoting metacognitive development (5).

In the context of VS, the knowledge platform widely used in clinical education is CyberpatientTM, developed by the Department of Surgery at the College of British Columbia. This interactive educational approach is grounded in problem-based learning and the development of clinical decision-making abilities (6). The results of the study conducted by Farahmand (7) show that the use of the CyberpatientTM virtual patient simulator is considered effective in improving clinical skills, as well as in the teaching-learning process of students, resulting in an increase in the effectiveness of clinical education and a reduction in costs (7).

The digital patient allows the student to interact directly with the simulator target, which are fictitious patients, known as avatars, with specific cases for each curricular unit. This structure enables immersive learning, as well as high student commitment and participation (8, 9). Because the interactions promoted by VS encourage student participation in learning by means similar to face-to-face practices (10).

There are other virtual platforms available on the market with features that allow the virtual environment to be used with multi-user participation. These functions provide space for instant reactions and feedback, as well as being more flexible in the formulation of scenarios, which makes the learner feel comfortable, and the reuse of virtual objects which reduces the cost of building scenarios (10).

Users can create their own avatars and design spaces and tasks according to situational demands. This creates global communication between users of these platforms, making it possible to share multiple experiences based on the real world and thus overcome the time and space limitations of learning methods. In this sense, the possibilities of multi-user virtual environments, such as Second Life® and others, have been considered the latest generation of educational techniques (5).

In order to explore the context of virtual simulations, a preliminary scientific search was carried out in April 2023 to identify possible existing scoping reviews on the topic in question. The following information sources were consulted: Joanna Briggs Institute, Cochrane Library, Web of Science, PubMed, Prospero and Open Science Framework. However, the search did not identify any review studies or protocols using clinical simulation with cybernetic patients in the teaching-learning process for nursing students.

Although digital technologies for health education have progressed substantially, undergraduate nursing programs still encounter significant barriers, including restricted opportunities for clinical placements, concerns regarding patient safety, and limitations in both human and material resources. These challenges have been further exacerbated by growing student cohorts and interruptions to in-person training activities, particularly during the COVID-19 pandemic.

Virtual simulation has gained prominence as an innovative educational approach to overcome these challenges, especially through digital patient–based simulations that employ interactive, screen-based virtual patients to enhance clinical reasoning, decision-making, and communication skills. Nevertheless, despite their increasing adoption, the use of digital patient simulations in nursing education remains heterogeneous, and the evidence concerning their effectiveness, scope, and limitations is dispersed.

Currently, there is no comprehensive synthesis that systematically maps how digital patient simulations are used in nursing education, the competencies they address, and the methodological approaches employed to evaluate their outcomes. This gap limits evidence-informed decisions regarding the integration of these digital technologies into nursing curricula.

In light of the growing integration of digital health tools into professional training, it becomes essential to systematically identify and consolidate the available evidence regarding the application of clinical simulation with digital patients in nursing education. Simulations involving digital patients constitute an innovative intersection of health informatics, educational technologies, and clinical skills development, and are consistent with core principles of digital health.

A scoping review is especially appropriate given the conceptual and methodological diversity of the field and the emergent nature of the evidence. By synthesizing study characteristics, educational aims, technological attributes, and reported learning outcomes, this review will map the current knowledge base and highlight existing gaps.

The findings may support educators, institutions, and policymakers in evidence-informed curriculum development, guide future research, and contribute to discussions on the role of digital technologies in strengthening clinical competence, communication, and patient safety in nursing education.

Accordingly, this study seeks to present a scoping review protocol designed to systematically identify and map the existing body of evidence on the application of clinical simulation with digital patients within the teaching–learning process of nursing students.

## 2 Methods and analysis

**Figure 1:**
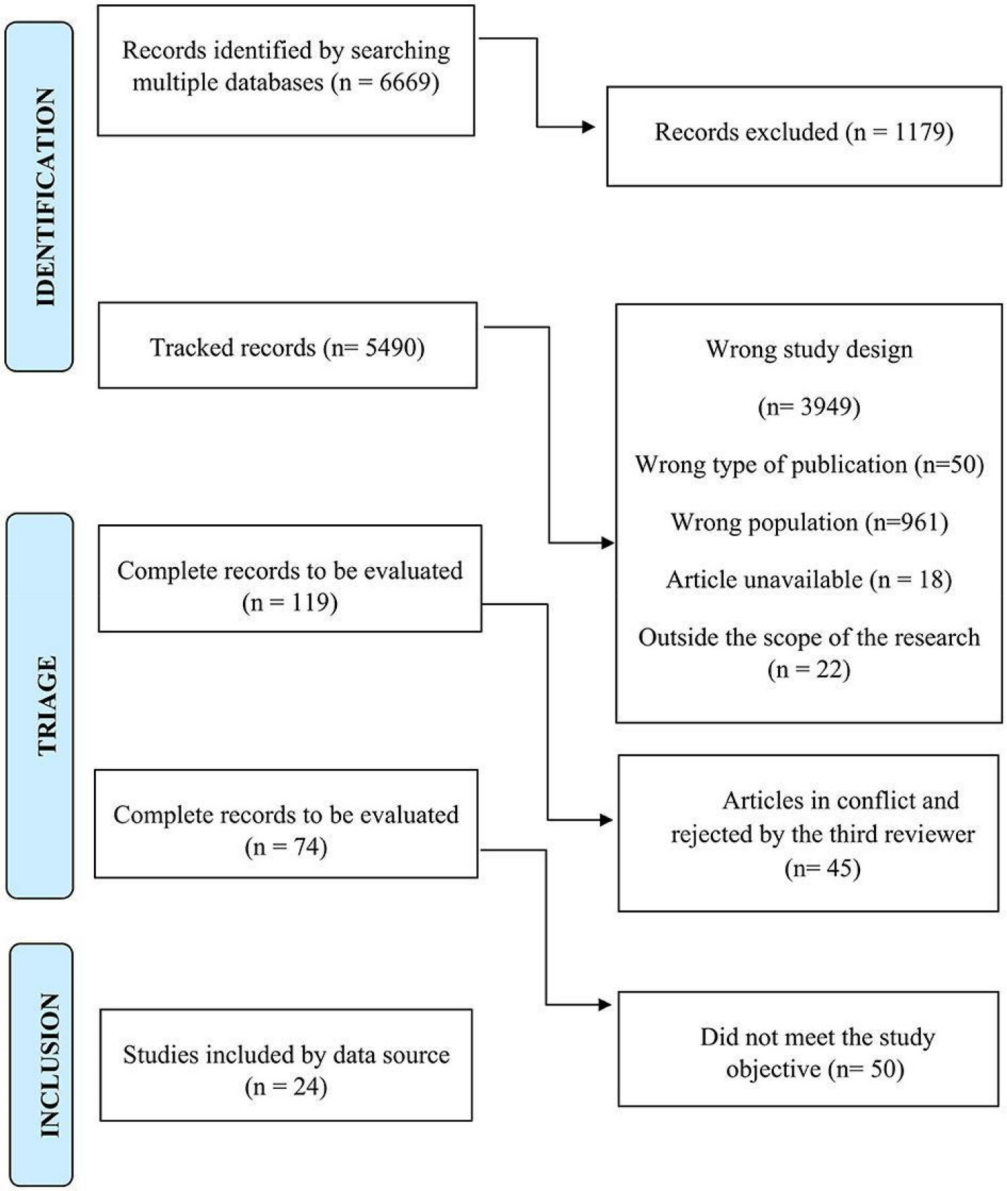
PRISMA-ScR flowchart showing the selection of sources of evidence. Source: Developed by the author (2025).

Source: Current research, 2025.

This scoping review protocol is based on the theoretical framework proposed by Peters et al. (11) and will be developed in accordance with the methodological guidance issued by the Joanna Briggs Institute (JBI) and guided by the Preferred Reporting Items for Systematic Extension of reviews and Meta-Analyses extension for Scoping Reviews (PRISMA-ScR), as illustrated in Figure 1. The protocol was registered with the Open Science Framework (OSF) under DOI number 10.17605/OSF.IO/GAXR6.

### 2.1 Patient involvement

As this is a scoping review protocol, there was no direct relationship with the patients involved, as previously carried out studies were used as the basis for this protocol.

Step 1. Define and align the research objectives and questions

To define the research question, the PCC mnemonic was used, in which the Population (P) - Nursing students; Concept (C) - Clinical simulation with cybernetic patient; and Context (C) - Teaching-learning process. Thus, the guiding question of the review is: What evidence exists regarding the application of clinical simulation involving digital patients in the teaching–learning processes of nursing students?

Step 2. Definition and alignment of eligibility criteria in accordance with the study objectives and research questions

Sources with full-text availability will be considered eligible, including peer-reviewed articles, dissertations, and theses that examine the academic training of nursing students through the use of clinical simulation with digital patients. No restrictions will be applied regarding geographic location, publication period, or language. When required, translation support will be sought to ensure accurate data extraction.

Reflection articles, editorials, review studies (integrative review, scoping review, systematic review and meta-analysis) and research that does not cover the health area will be excluded.

Step 3. Description of the search for evidence, selection, data extraction and presentation of evidence

The search process will be implemented in three sequential phases, in accordance with the methodological recommendations proposed by Peters et al. (11), namely:*First stage - Identifying descriptors and keywords:*

At this stage, an exploratory database searches were performed in PubMed and the Virtual Health Library (VHL) databases to determine the primary Medical Subject Headings (MeSH) and Health Sciences Descriptors (DeCS) relevant to the subject area.

To ensure comprehensive identification of relevant evidence across multiple databases, the search strategy was developed using a combination of controlled vocabularies, including DeCS and MeSH, along with free-text terms and database-specific subject headings. An initial filter was applied to exclude studies involving animals. Subsequently, additional synonyms and keywords were identified to refine the strategy. The search approach was then reviewed, validated, and optimized in collaboration with an experienced librarian. The finalized search strategy was constructed for MEDLINE via PubMed and is presented in Table 1.

**Table 1:** Search strategy for a scoping review mapping the available evidence on the use of clinical simulation with digital patients in teaching and learning processes among students and health professionals.

#	Searches
1	“Simulation Training”[Mh] OR “Interactive Learning”[title] OR “Patient Simulation”[Mh] OR “High Fidelity Simulation Training”[Mh] OR Telesimulation[title] OR “Virtual simulation”[title] OR ciberpatient[text word] OR ciber-patient[text word] OR “computer simulation”[title] OR “Computer-assisted learning”[title]
2	Concept A - This line provides the terms for the Digital Patient group #1
3	Students[Mh] OR Student*[title] OR “Students, Nursing”[Mh] OR “Students, Nursing”[tiab] OR “Pupil Nurse”[tiab] OR “Nursing Student*”[tiab]
4	Concept B - This line provides the terms for the Nursing Students group #3
5	“Training Technique”[title] OR Teaching[Mh] OR Teaching[title] OR “Teaching Method”[title] OR “Academic Training”[title] OR “Educational Technic*”[title] OR Learning[Mh] OR Learn*[title]
7	Concept C - This line provides the terms for the Teaching/Learning group #6
8	This line Combines A and B and C (3,061 records)
9	Animals[Mh] NOT humans[Mh]
10	8 NOT 9 This line provides the result of A and B and C without animal studies (3,057) records)

### 2.2 Second stage - database definition for data collection

Relevant studies were identified through searches conducted in multidisciplinary electronic databases within the health sciences, as well as in repositories of theses and dissertations. Peer-reviewed literature was retrieved from MEDLINE/PubMed, Embase, Web of Science (WoS), the Education Resources Information Center (ERIC), and the Cumulative Index to Nursing and Allied Health Literature (CINAHL). Gray literature was explored using national and international repositories, including the Catalogue of Theses and Dissertations of the Coordination for the Improvement of Higher Education Personnel (CAPES), the Electronic Theses Online Service (EthOS), the Open Access Scientific Repository of Portugal (RCAAP), the National ETD Portal, Theses Canada, the Portal de Tesis Latinoamericanas, and WorldCat Dissertations and Theses.

### 2.3 Third stage - identification of additional sources through reference list screening

The reference lists of publications included during the data extraction phase will be examined to identify additional eligible sources that may not have been captured through the initial search strategy. When necessary, the corresponding authors of included studies will be contacted via email to obtain Supplementary Information.

### 2.4 Fourth step: final database search strategy

With the help of a librarian, a search strategy was devised to retrieve studies from databases, as well as national and international grey literature sources. To do this, controlled vocabularies (DeCS and MeSH), text words, and subject headings were used. Finally, a filter was applied to exclude animal studies. The complete search strategy is presented in Table 2.

Step 4. Searching for evidence

**Table 2:** Search strategy initially used in databases.

Database	Search strategy	Results	Date
PUBMED	((“Simulation Training"[MeSH Terms] OR “Interactive Learning"[Title] OR “Patient Simulation"[MeSH Terms] OR “High Fidelity Simulation Training"[MeSH Terms] OR “Telesimulation"[Title] OR “Virtual simulation"[Title] OR “computer simulation"[Title] OR “Computer-assisted learning"[Title]) AND (“Training Technique"[Title] OR “Teaching"[MeSH Terms] OR “Teaching"[Title] OR “Teaching Method"[Title] OR “Academic Training"[Title] OR “educational technic*"[Title] OR “learning"[MeSH Terms] OR “learn*"[Title]) AND (“students"[MeSH Terms] OR “student*"[Title] OR “students, nursing"[MeSH Terms] OR “students nursing"[Title/Abstract] OR “Pupil Nurse"[Title/Abstract] OR “nursing student*"[Title/Abstract])) NOT (“animals"[MeSH Terms] NOT “humans"[MeSH Terms])	3.547	*20/03/2025*
WEB OF SCIENCE (WOS)	*TS* *=* *(“Simulation Training” OR “Interactive Learning” OR “Patient Simulation” OR “High Fidelity Simulation Training” OR Telesimulation OR “Virtual simulation” OR ciberpatient OR ciber-patient OR “computer simulation” OR “Computer-assisted learning”) AND TI* *=* (Student* OR “Students, Nursing” OR “Pupil Nurse” OR “Nursing Student*”) AND TI *=* (“Training Technique” OR Teaching OR “Teaching Method” OR “Academic Training” OR “Educational Technic*” OR Learning) TOPICO (“Simulation Training” OR “Interactive Learning” OR “Patient Simulation” OR “High Fidelity Simulation Training” OR Telesimulation OR “Virtual simulation” OR ciberpatient OR ciber-patient OR “computer simulation” OR “Computer-assisted learning”) AND TÍTULO (Student* OR “Students, Nursing” OR “Pupil Nurse” OR “Nursing Student*”) AND TÍTULO (“Training Technique” OR Teaching OR “Teaching Method” OR “Academic Training” OR “Educational Technic*” OR Learning) (Título)	1.147	*20/03/2025*
*EMBASE*	(’simulation training':ti,kw,ab OR ‘interactive learning':ti,kw,ab OR ‘patient simulation':ti,kw,ab OR ‘high fidelity simulation training':ti,kw,ab OR ‘simulation-based training':ti,kw,ab OR telesimulation:ti,kw,ab OR ‘virtual simulation':ti,kw,ab OR ciberpatient:ti,kw,ab OR ‘ciber patient':ti,kw,ab OR ‘computer simulation':ti,kw,ab OR ‘computational simulation':ti,kw,ab OR ‘computer-based simulation':ti,kw,ab OR ‘computer-assisted learning':ti,kw,ab) AND (‘nursing student':ti,kw,ab OR ‘nurse student':ti,kw,ab OR ‘pupil nurse':ti,kw,ab OR nurs*:ti,kw,ab) AND (‘training technique':ti,kw,ab OR teaching:ti,kw,ab OR ‘teaching method':ti,kw,ab OR ‘academic training':ti,kw,ab OR ‘educational techn*':ti,kw,ab OR learning:ti,kw,ab OR ‘learning situation':ti,kw,ab OR ‘knowledge acquisition':ti,kw,ab) AND [embase]/lim	725	20/03/2025
*ERIC*	*descriptor:“Computer Simulation” OR descriptor:“Computer Assisted Instruction” AND descriptor:“Nursing Students” OR descriptor:“Nursing Education” OR nursing AND descriptor:Learning OR Instrution*	392	20/03/2025
*CINAHL*	(SU “Simulation Training” OR AB “Simulation Training” OR SU “Interactive Learning” OR AB “Patient Simulation” OR SU “Patient Simulation” OR SU “High Fidelity Simulation Training” OR AB Telesimulation OR SU “Virtual simulation” OR TI ciberpatient OR SU ciber-patient OR MH “computer simulation” OR SU “computer simulation” SU “Computer-assisted learning”) AND (MH Students OR MH “Students, Nursing” OR SU “Pupil Nurse” OR SU “Nursing Student*” OR MH nurs*) AND (SU “Training Technique” OR TI Teaching OR MH “Teaching Methods” OR AB “Academic Training” OR SU “Educational Technic*” OR MH Learning OR SU “Learnng Methods”)	412	20/03/2025
Gray literature			
CAPES Catalog of Theses and Dissertations	*“Clinical simulation” OR ‘Patient Simulation’ AND “Nursing”*	26	20/03/2025
EthOS	*clinical simulation AND nursing*	61	20/03/2025
RCAAP	*clinical simulation AND nursing*	17	20/03/2025
National ETD Portal	*title:"clinical simulation” AND nursing*	15	20/03/2025
Theses Canada	*clinical simulation AND nursing*	76	20/03/2025
Portal de Tesis Latinoamericanas	*"clinical simulation” OR “Patient Simulation”) AND (nursing)*	170	20/03/2025
World Cat Dissertations and Theses	*"clinical simulation” AND “nursing” AND “Learning"*	1.027	20/03/2025

Source: Current research, 2025.

ERIC, Education Resources Information Center; CAPES, Coordenação de Aperfeiçoamento de Pessoal de Nível Superior; EthOS, Electronic Theses Online Service; RCAAP, Portugal's Open Access Scientific Repository.

Once the studies have been collected, they will be identified, grouped and entered into the free Web Rayyan software (12) to manage the paired screening of studies based on titles and abstracts, using the inclusion and exclusion criteria established, guided by this protocol, in blinded mode between the reviewers to classify them as included and excluded, in addition to removing duplicate studies. In the event of conflicts, consensus will be sought and, if there is still disagreement, the final decision will be made by a third reviewer.

Step 5. Selection of evidence

Following the screening of titles and abstracts, records deemed potentially eligible will have their metadata imported into a Microsoft Excel® (2021) database. Full texts will then be independently assessed in pairs to determine final inclusion or exclusion. All decisions related to study selection, including eligibility criteria and reasons for exclusion, will be systematically documented and presented in a dedicated flow diagram in accordance with PRISMA-ScR guidelines (13).

Step 6. Data extraction

A data extraction instrument will be developed in alignment with the objectives and guiding research question of this review. Extracted data will be recorded in a Microsoft Excel® (2021) spreadsheet. Table 3 presents a proposed template for data extraction, developed as a framework to guide this review and to support future authors conducting studies with similar objectives.

Step 7. Evidence analysis

**Table 3:** Data extraction tool.

Article identification:	
Type of publication:	
Year and Country:	
Formation of the first carr:	
Objective:	
Methodological design:	
Population	
Target audience:	
Level of training:	
Concept	
Cyberpatient concept:	
Theme and purpose of the technology:	
Technology validation:	
Context	
Impact of the use of technology:	
Limitations to the use of technology:	

Source: Authors, 2025.

The results will be analyzed according to the research objective in order to obtain a quantitative and qualitative analysis and presented in a flowchart. For the quantitative analysis, the data were examined using statistical analysis performed with the Statistical Package for the Social Sciences (SPSS), version 24.0 will be used to present descriptive statistics with absolute frequency and percentages. For the qualitative analysis, thematic analysis will be used due to its flexibility in identifying patterns in research questions and will allow the impacts and limitations of the use of technology to be categorized (14). Finally, this data will be discussed on the basis of the literature and the research objectives and questions.

Step 8. Presentation of results

Extracted data will be synthesized and displayed using descriptive tables and flow diagrams, consistent with the objectives and guiding research question of the review. Findings will be comprehensively reported through a narrative synthesis, following the PRISMA-ScR reporting standards as outlined by Tricco et al. (15)

Step 9. Summary of the evidence, conclusions and implications of the findings

With the outcome of the previous stages, a summary of the results of the scoping review linked to the objective of the study will be carried out. In this way, its conclusion will be substantiated and presented, highlighting the gaps in knowledge found and providing guidelines for future studies.

### 2.5 Ethics and dissemination

No primary data will be generated in this study; therefore, approval from a formal ethics committee is not necessary. Study findings will be shared through peer-reviewed journals, presented at scientific conferences, and disseminated via relevant email lists and social media channels. The findings of this study may guide future research aimed at addressing existing evidence gaps in the field.

## 5 Conclusion

The evidence suggests that simulation involving digital patients supports the progressive development of nursing students’ confidence through repeated practice. Moreover, simulation-based scenarios offer expanded opportunities for learners to refine clinical skills and to assume a more active role in clinical decision-making.

Virtual patient simulation also provides a safe learning environment in which errors can be explored without jeopardizing patient safety. Core elements of the teaching learning process such as the enhancement of critical thinking, learner satisfaction, and self-confidence were consistently reported across the studies. From an economic perspective, this instructional approach was identified as a more cost-effective alternative when compared with mannequin-based simulation.
